# Development of Cultural and Environmental Awareness Through Sámi Outdoor Life at Sámi/Indigenous Festivals

**DOI:** 10.3389/fspor.2021.662929

**Published:** 2021-07-15

**Authors:** Bente Ovedie Skogvang

**Affiliations:** INN/Inland Norway University of Applied Sciences, Elverum, Norway

**Keywords:** Sami festivals, outdoor activities, sustainability, cultural capital, indigenous festivals

## Abstract

The indigenous people Sámi are an ethnic minority living in Finland, Norway, Russia, and Sweden. Throughout history, Sámis have been living close to nature. Working with reindeer husbandry, fishing-farming, hunting, herding, and harvesting for food supplies, has traditionally been an integral part of their lives. Currently, only 2,500 of the ~65,000 Sámis in Norway are operating reindeer husbandry (2019). Most Sámis today work in mainstream jobs, and the fishing-farming culture gradually become more like the mainstream societies where Sámis live. Fieldwork with participant observation and semi-structured interviews carried out at Riddu Riđđu Festivala in the period 2009–2018. In addition, the governing bodies of seven other Sámi festivals have been interviewed. All together 46 in-depth interviews and participant observations conducted, in addition to document analysis of the festivals. The aim was to study how physical and outdoor activities included in the festivals create indigenous people's identities and cultural understanding and how the activities at the festivals might develop climate and environmental awareness. Indigenous festivals and their governing bodies offer many different forms of physical and cultural activities from Sámis and different indigenous peoples to the youth and children taking part. Further, the study shows that important aims for the organizers are to spread the knowledge about Sámis (i.e., local coastal Sámis and regional reindeer/Inland Sámis) and other indigenous peoples, and making environment-friendly festivals. They are trying to educate the children and youth in the cultural practices of their forefathers and foremothers. The manifold of activities offered at the festivals seem to create sustainable ties between persons, which equip the participants with social and cultural capital in addition to networks across festivals organizations internationally. The participants further express that taking part in the festival activities create symbolic capital, due to that they might express their indigeneity at the festivals both for people living in the region and for a greater audience. According to the participants, the festivals have equipped the participants with cultural awareness, as well as the children and youth taught an appreciation of nature so they can enjoy and respect nature and develop climate and environmental awareness.

## Introduction

The article concerns how education through physical and outdoor activities offered to children and youth at Sámi/indigenous festivals in Norway might influence the participating children and youth. Viken and Jaeger (2012), Tjora, 2013, and Jæger (2019) argue that there is a lack of studies on festivals in Norway in general and on Sámi festivals in particular. Studies show that Sámi festivals in Norway create Sámi people's identities (Pedersen and Viken, 2009; Skogvang, 2013; Jæger, 2019). Other studies underline that the festivals are and have been crucial in the revitalization of the Sámi cultures and that they are important for development of places (Hansen, 2007; Viken, 2011; Viken and Jaeger, 2012). Jæger (2019) studied different kinds of festivals in Northern Norway, and she underlined the importance of the festivals as meeting places, as crucial in the development of tourism, as well as that festivals in Northern Norway are a benefit for local communities in the North. Although some studies are carried out, there are few studies on children and youth, and therefore, the aim of the overall study was to increase the knowledge about outdoor life and physical activities among children and youth taking part in Sámi festivals in Norway. How participation in Sámi/indigenous festivals in Norway might influence development of cultural and environmental awareness among young people is highlighted in this article. The governing bodies and participants at the international indigenous *Riddu Riđđu Festivala* and seven other Sámi festivals in Norway state that to participate at indigenous festivals creates indigenous peoples' identities, increases their cultural and environmental understanding and extends their knowledge in both Sámi and the culture of other indigenous peoples. *Friluftsliv* or outdoor life in Norway is a tradition with rituals that have been passed from generation to generation as “a lifelong communal process” (Dahle, 2007, pp. 23–24). In the four Sámi languages, there is no word for what Norwegians call *friluftsliv*, which might be translated to outdoor life. Instead, the Sámi languages use active words about doing, creating, and making things in nature. For example, to put up a “*lávvu*” (the Sámi tent) includes to erect the *lavvu*, furnish it and all other activities you are making in and around the *lavvu*, and it is called “*l*á*vostallat*” /”*lavvu*-ing” in Northern Sámi. “*Doallastat*” or “bonfire-ing” includes to collect wood, birch-bark, and make a bonfire, as well as all the activities around the fire. Despite the long tradition of outdoor life in the circumpolar area, there are few studies carried out about outdoor life from “insiders” with a Sámi perspective. When focusing on sustainability, a broad perspective is used. In line with United Nations' (2015) sustainable goals, where some of the UN goals are connected to culture and climate awareness, i.e., climate action, life on land and water, sustainable communities, education, strong institutions, and reduced inequalities, they are relevant for the festivals presented in this article.

How identity is articulated through outdoor activities and how indigenous peoples have used sports or physical activities to oppose mainstream society and engage in nation building are shown in international studies (Bale and Cronin, 2003; Mangan and Ritchie, 2004; Hallinan and Judd, 2013). Tan (2012) and Trollvik (2014) have studied indigenous traditions and gatherings in Taiwan. Both studies stress that a key factor for survival of a specific culture is the embodiment of cultural practices. For instance, through learning the Amis aboriginal song (Tan, 2012) or through taking part in indigenous festivals (Trollvik, 2014), the youth are taught traditional and practical knowledge, which build their self-esteem. Participation in indigenous cultural festivals in Australia is experienced as empowering as well as crucial for the community and for indigenous peoples' health and well-being (Phipps and Slater, 2010). Other comparable international events to the Sámi festivals in Norway, where indigenous peoples use these games in the revitalization process of indigenous peoples, are the *North American Indigenous Games* and the *World Indigenous Nation games* (Paraschak, 1997; Forsyth and Wamsley, 2006; King, 2006; Hallinan and Judd, 2013). The *Arctic Winter Games* (2021) celebrate sports, social exchange, and cultures; and in the postponed games (due to COVID-19) in 2023, the event celebrates its 50th anniversary. “*The Wood Buffalo 2023 Arctic Winter Games*” includes athletes from the Northwest Territories, Yukon, Nunavut, Alaska, Greenland, Russia (Yamal), Nunavik (Northern Quebec), Northern Alberta and the Sámis of Norway, Sweden, and Finland (Arctic Winter Games, 2021). Since 2004, several Sámi youth have participated and competed in the *Arctic Winter Games* with successful results especially in cross-country skiing (men and women) and indoor soccer/futsal (women).

Indigenous peoples in North America have similar experiences as Sámis about discrimination and assimilation (Paraschak, 1997; Forsyth and Wamsley, 2006). Paraschak (1997) analyzed how race relations in indigenous games in Canada are shaped and reshaped by “practical consciousness” of native peoples, and Forsyth and Wamsley (2006) emphasized how the world Indigenous Nations' games and the North America indigenous games are used as cultural resistance. The indigenous leaders in Canada have managed to reverse the disempowerment that occurred during the early years of the events and have instead managed to use these games to empower indigenous peoples of Canada. Similar to the Arctic Winter Games, these games aim to establish an international network of indigenous peoples within sports, and they have succeeded (Forsyth and Wamsley, 2006). Heine and Scott (1994) studied how traditional games of the Dene (Athapascan Native Americans) of Northern Canada were played in a similar format as the modern Olympic Games. They conclude that these festivals have provided the Dene with an opportunity to actualize the cultural importance of their old games within the context of contemporary Euro-Canadian culture. Tan (2012) showed how indigenous festivals on Taiwan, i.e., harvest festivals, are important sources of local pride in most indigenous communities, as they gather former residents, big families, friends, and tourists, all for a great celebration of the particular indigenous traditions. The author focuses on how the festivals on Taiwan are strengthening the communal relationships, and they also give possibilities of reconnecting to the indigenous part of the participants' identity, a connection that they often do not express very explicitly because they are living in the city (Tan, 2012). Does knowledge learned through Sámi/indigenous festivals in Norway create sustainable societies and cultures, equip the participants with valued symbolic capital and develop environmental and cultural awareness?

Firstly, I briefly present the context, the history and the participation of indigenous peoples of the festivals studied. Then, I give a brief presentation of the theoretical perspectives. I adapt Bourdieu's theory of social space, capital, and habitus as tools when I analyze the data. Thirdly, methods are presented, consisting of a fieldwork (participant observation and semi-structured interviews) at the Riddu Riđđu festival, interviews with the governing bodies of seven other Sámi festivals in Norway and document analysis of the same festivals. In addition, I reflect upon the ethical considerations and my position as a coastal Sámi writing from an insider/outsider Sámi perspective. Fourth, some of the results are introduced and discussed in four sections;

- symbolic capital through Sámi (*doallastat, lávostallat, stallu*, and *goatsuballu*) and indigenous outdoor activities;
- Sámi/indigenous festivals—making indigeneity stronger;
- “*The Northern people of the year*” at Riddu Riđđu—creating cultural awareness; and
- environmental awareness through “Miljøfyrtårn” and the “*Indigenous Climate Camp*.”

Finally, I conclude that taking part in Sámi/indigenous festivals in Norway is empowering and imparts participating indigenous and non-indigenous peoples with knowledge and skills of both minorities and the major populations, creates networks and sustainable ties between persons and institutions, and inspires children and youth through learning skills in nature, to enjoy the life in nature, care about the environments and respect both nature and other peoples' cultures.

### The Context: Sámi in Four Countries—Who Are We?

The indigenous peoples *Sámi* are an ethnic minority living in Finland, Norway, Russia, and Sweden, with their own history, culture, language, and settlement areas (Hætta, 2007), but today, Sámis live in both rural and urban areas of their countries. Sámis have lived in the Nordic Peninsula as far back as can be traced and long before the nation states were established (Solbakk, 2006). Through history, Sámis have been living close to nature, and their work have been reindeer husbandry, fishing–farming, and hunting, herding, and harvesting for food supplies, which traditionally have been a crucial part of their cultures. Today, Sámis work in both traditional and mainstream jobs and live all over in the four countries, but most of them in the Northern parts. The Sámi peoples are accepted as indigenous peoples in all four countries. Finland, Norway, and Sweden have a *Sámi Parliament; Sámediggi/Sametinget*. Despite the similarities, there are substantial differences (Skogvang, 2017). The Sámi Parliament in Norway (established in 1989) has the collective right in acting as an advisory body to the Norwegian Parliament, *Stortinget*, and might participate in any governmental Sámi projects and plans. In 1990, Norway was the first country to ratify the “*ILO Convention 169 concerning Indigenous and Tribal Peoples in Independent Countries*” (International Labour Organization, 1989). By Article 32, Sámi rights are secured because Norway is bound to cooperate across states and borders. Due to that, the convention is not ratified in the three other countries; Sámis in Norway have stronger collective rights (Skogvang, 2017, pp. 40–41).

In Norway, Section § 2–6 of “*The Sámi Act*” (Government, 1987) defines Sámis as “All persons who make a declaration to the effect that they consider themselves to be Sámi, and who either (a) have Sámi as their domestic language, or (b) have or have had a parent, grandparent or great-grandparent with Sámi as his or her domestic language, or (c) operating reindeer husbandry.” The estimates of the Sami population vary in accordance with criteria used like genetic heritage, mother tongue, and the personal sense of ethnicity (Broderstad, 2008). Today, there are a total of between 81,000 and 111,000 Sámis, with roughly 50,000–65,000 living in Norway (Skogvang, 2017). Of Norway's 5.3 million inhabitants, 18,103 are enrolled in Norway's Sámi electoral register (Sàmediggi/Sametinget, 2019). However, only 2,500 of the ~65,000 Sámis in Norway are operating reindeer husbandry (2019), which means that most of the Sámi today work in similar jobs as the majority population (Regjeringen, 2018–2019). The assimilation through the “*Norwegianization process*” from the 1850s until recent time has been a menace to Sámi cultures inclusive of the Sámi languages, and due to the pressure of assimilation, not all Sámis want to show their ethnic identity (Skogvang, 2019). The four resisting Sámi languages are vulnerable, with the Northern Sámi as the largest and the Southern Sámi as the most vulnerable. According to the Sámi Language Council, there are only 25,000 Sámi language users left in Norway, due to the fact that the language has been forbidden to be used for a long time, which resulted in the Sámi no longer being the everyday language in many families (Sàmediggi/Sametinget, 2019).

To take part in festivals gives free spaces to live out enjoyment and recreation, where tradition, customs, myths, beliefs, and freedom are celebrated (Falassi, 1987). These celebrations are identity markers (Quinn, 2005; Jæger, 2019) and social institutions that started in ancient times (Hegnes, 2006; Hætta, 2007; Tjora, 2013). The Sámi gatherings in Norway have similar long traditions (Hætta, 2007) but were defined as festivals in recent times. The oldest one, the Easter festival in Guovdageadnu/Kautokeino, which also includes the Sámi song contest “Melody Grand Prix,” celebrates its 50th anniversary in 2021. Easter celebrations have been crucial for Sámis much longer than that, when the reindeer herding Sámi families came back to their villages for celebrations and reunions with their relatives and families as well as cultural and trading exchanges with the local coastal Sámi families (Hætta, 2007). Several of the festivals are still organized during Easter or during summer holidays or in connection with the Sámi National Day every February 6. The *Riddu Riđđu Festivala* (Riddu Riđđu, 2019a) is another large Sámi festival in Norway, which was started by young coastal Sámis around a fireplace in Olmáivággi/Manndalen (Sápmi/Norway) in 1991, with the aim of taking back their lost coastal Sámi culture (Hansen, 2007). Riddu Riđđu means “a little storm along the coast” (Hansen, 2007), and the festival celebrates its 30th anniversary in 2021.

Earlier studies of Riddu Riđđu show the importance for the revitalization of Sámi culture as well as development of places, i.e., economic development in the region and the municipalities where the festivals are situated (Hansen, 2007; Viken and Jaeger, 2012). The festivals studied are situated in the northernmost part of Norway, where the majority population are Sámi (between 60 and 80%). In the municipality Gaivuotna/Kåfjord, where the Riddu Riđđu festival is held, 60% (of 2,074) and in the festival village, Olmáivággi/Manndalen (Sápmi/Norway), 80% (of 850) are Sámi (Statistisk Sentralbyrå, 2019). In Eriksen (2002) terms, this is an area of “cultural complexity” due to many peoples living in the region. Hovland (1999) elaborated that it is crucial to understand the many different ethnic groups in the region and the influence of the assimilation policy, which have led to shame and low self-esteem among young people in the area where the festivals are situated. On June 12, 1987, the Sámi Act came into force in Norway. In spite of that, recent studies show that Sámis still struggle with experiences of inferiority, harassment, and discrimination in Norway (Eythórsson, 2003; Hansen, 2011, 2015; Hansen and Skaar, 2021).

### Theory

How knowledge learned through Sámi/indigenous festivals in Norway might create sustainable societies and cultures and educate children in Sámi cultures, language, and outdoor life as well as environmental and cultural awareness is studied by applying Bourdieu's theories of *social space, symbolic capital*, and *habitus*. Social space refers to the overall society where actors stand in positions relative to each other and where the positions structure the actors' preferences and access to particular fields (Bourdieu and Wacquant, 1992; Bourdieu, 1993). The Sámi/indigenous festivals are autonomous and independent of other fields while simultaneously being dependent of the surroundings and other parts of the society around in the implementing of the festivals, i.e., local authorities in the region and the municipality, Sámi language centers, schools, universities, museums, libraries, media, handcraft organizations, sports clubs, and outdoor life organizations.

One of the central concepts in Bourdieu's theory is *capital*, and he puts forward different types of capital; economic, cultural, social, symbolic, and field-specific capital. A field is composed of actors struggling for the types of capital recognized in the field, which creates positions (of power) in the field. The structure of a field is defined by the distribution (amount of composition) of capital, which is based on the result of previous struggles and which directs future struggles (Bourdieu and Wacquant, 1992; Bourdieu, 1993). Bourdieu defines a field as:

“… positions, including those of power, which have developed during history and which create structures between the positions of actors struggling for the field-specific capital and the fields' hegemony and the right to define its rules of function and change; and the habitus of the agents, their systems of dispositions which are based on objective material and social conditions as well as subjective preferences.” (Bourdieu and Wacquant, 1992, pp. 104–105)

Bourdieu (1996) applied *symbolic capital* to analyze that some people or institutions, exams or titles, piece of art or scientific work get huge trust and reputation and are recognized as valuable and superior to others. “Symbolic capital is any kind of characteristic (any kind of capital; physical, economic, cultural, or social) which are perceived of social actors, with perception categories as is possible to recognize it and value it” (Bourdieu, 1996, p. 61). In other words, symbolic capital is what social groups are identified with, appreciated as valuable and creditable or worthy. One dimension of symbolic capital is ethnic identity, which function as a positive or negative symbolic capital (Bourdieu, 1996, p. 90). Furthermore, Bourdieu (1996) more specifically explained that cultural capital exists in three forms: the embodied knowledge (system of dispositions or habitus), the objective compositions (i.e., art, paintings, library, books, and tools) and the institutionalized compositions (i.e., exams, education titles and work titles). Symbolic capital is a key concept by Bourdieu (1993, 1996), which is useful in analyzing how some humans or institutions, exams or titles, art or scientific work gain huge trust, reputation and prestige and are appreciated as honorable, truthful, and superior to others.

Values and resources constitute an individuals' capital and the objective part of the structures, and *habitus* is a socially constructed system which that acquired knowledge through praxis and is constantly oriented toward practical functions (Bourdieu and Wacquant, 1995). Bourdieu (1995, p. 219) defined habitus as an individuals' “embodied, social structures” where the actors in their daily practice are subjects constructing the social world. Habitus might not explain behavior without relating to the context, and because individuals steadily have to respond to new contexts, changes in habitus occur (Bourdieu and Wacquant, 1995). The majority populations' dominant role in society is based on symbolic power and is taken for granted (doxa). Hovland (1999) studied young peoples' habitus in the circumpolar area of Norway and their experience of their own identity. He concluded that young peoples' identities in the region are marked by the “Norwegianization process,” with the assimilation and discrimination of Sámis and other ethnic minorities during the strong assimilation from the Norwegian government. He underlines that the young people and their cultural habituses are characterized by the discriminating history from the mainstream society.

Today, Norway is founded on the territory of two peoples: Sámis and Norwegians (Skogvang, 2019). But through the “*Norwegianization process*” with assimilation and discrimination, Sámis in Norway have been suffering from oppression from the majority cultures for a long time (Skogvang, 2017). Skogvang (2019) presented how the governing bodies in Norway forced the Sámis to become Norwegians through assimilation during more than 100 years from circa 1850. These experiences have created the indigenous peoples of Norway's habituses for ages, which are similar to indigenous peoples' experiences worldwide. Hovland (1999) found that experiences of discrimination and experiences of painful assimilation are deeply rooted in and fundamental to the habitus (Bourdieu and Wacquant, 1995) of the young Sámi in the region. Studies of indigenous peoples at other continents highlight similar experiences (Paraschak, 1997; Forsyth and Wamsley, 2006; King, 2006; Hallinan and Judd, 2013). Through learning skills and competences at the festivals studied, Sámi children and youth might turn a painful history to proudness during revitalized habituses, where their ethnicity is given value both on and off the festival stages. To be taught such Sámi and/or indigenous cultural capital, as well as capital sourced to dominant social spaces from non-indigenous activities, assists individuals in realizing their cumulative advantage and may be associated with improved educational outcomes, i.e., knowledge and skills in Sámi culture and language, skills in nature, knowledge of minority and majority cultures, climate competence, and self-esteem.

## Methodology

### Data Collection Through Fieldwork and Semi-Structured Interviews

Fieldwork was carried out yearly in the period 2009–2018 during the festival week in July at Riddu Riđđu. Data collection was conducted by participant observation and in-depth interviews at this festival, along with similar interviews with the governing bodies of seven other Sámi festivals in Norway. In addition, a document analysis is conducted of the festival's strategy documents, reports, and websites for Riddu Riđđu and seven other Sami festivals in Norway. Observation at Riddu Riđđu focused on activities at the Children's Festival *Mánáidfestivála* (for 3- to 13-year-olds) and *Nourat* (for 13- to 18-year-olds). The participant observation was fulfilled during daytime, when the children's activities were held with field notes written in pauses, during lunchtime or after the daily activities. A semi-structured interview guide with open-ended questions based on the topics of the research questions was used to explore the informants' experiences and views on the festival. Topics addressed in the interviews are types of outdoor activities offered at the festival, aims of the activities, experiences of the activities and the activities' meaning for the participants, including the participants' experiences of the meaning for creation of identities, cultural and environmental awareness. The comprehensive data collection consists of 46 in-depth interviews, field notes from participant observations and document analysis; and they were carried out to contemplate how outdoor activities included in the festival create identity and awareness of nature and culture among Sámis and other indigenous and non-indigenous peoples participating at the festivals.

### Sample

A strategic selection (Patton, 2015) of informants is carried out through recruitment of participants who could describe their experiences relevant for the research questions asked about the Sami/indigenous festivals. The largest data material is from the Riddu Riđđu festival, where 39 in-depth interviews were carried out with 21 women/girls and 18 men/boys. Some of these informants are interviewed several times. In addition, seven in-depth interviews were carried out with the governing bodies (three men and four women) of other Sami festivals in Norway (see **Table 2**). Parents/guardians and children and youth below 18 years who took part in Riddu Riđđu are interviewed together. In addition, some adults (including some parents/guardians) are interviewed alone. The length of the interviews is from 45 to 120 min. The longitudinal design of the study of Riddu Riđđu gave thick descriptions. The comprehensive data collection enabled the researcher to study trends in the development of the activity provision as well as following up and interviewing four families at several Riddu Riđđu festivals. The adult interviewees are many and various from governing bodies, staff members, volunteers, performers, artists, non-indigenous participants, yearly indigenous guest peoples (“Northern People of the year”) and persons from the region with critical views on the festival. I conducted most of the interviews in Norwegian. When interviewing the “Northern People of the year,” I interviewed in English. When it was necessary, I used the limited Sámi words I know when I was speaking with Sámi children and youth, for instance, from Finland and Russia.

### Positioning and Reflexivity

Indigenous researchers such as the Sámi researcher Jelena Porsanger has contributed with an alternative to the Western paradigms. Porsanger (2004, p. 105) argued that we might “… contribute to the body of knowledge of indigenous peoples about themselves and for themselves, and for their own needs as peoples, rather than as objects of investigation.” In “*Decolonizing Methods*,” Maori Linda Tuhiwai Smith draws attention to “… centring our concepts and worldviews and then coming to know and understand theory and research from our own perspectives” (Smith, 1999, p. 91). Other indigenous researchers underline that collectivism and to think about nature and society as a whole built on collaboration and partnership are of large importance in indigenous peoples' societies and in the power between generations, nature and genders (Kuokkanen, 2000; Jaimes Guerrero, 2003). Rigney (1999, p. 119) stressed that “the interests, knowledge and experiences of indigenous peoples must be at the center of research methodologies and construction of knowledge about indigenous peoples.” Olsen (2016) underlined the need for research on indigenous peoples' premises, supporting indigenous peoples' interests as well as using indigenous peoples' own language as much as possible and pinpointing ideals such as respect, mutuality, humbleness and a relational approach.

The described “*Norwegianization process*” (Hovland, 1999; Skogvang, 2019) and experiences from the past have resulted in a skeptical view on researchers from the outside, which are comparable with experiences from other indigenous peoples, i.e., Maori and Aborigine (Smith, 1999). As a result of the “Norwegianization process,” I do not speak my parents' language Northern Sámi fluently, but I have an extended knowledge of the region where the festivals are situated. For instance, when I contacted the festival organizers, they were having doubt about giving me access to study the festival and said: “… several researchers and studies of the Sámis have been without understanding of our background and our lived experiences in the past, so we are sometimes skeptical to researchers from outside.” Anyway, due to that I am a Sámi myself, the answer at last was: “… you belong to our people, and therefore we trust you.” However, the Sámi cultures are numerous from Inland Sámis with reindeer husbandry to Sámis living in the forest, in the mountains, along the long coastline of Norway, as mentioned in urban areas with similar work as the mainstream population. I am a coastal Sámi and carried out fieldwork in the rural area where I lived the first 16 years of my life and where my father still lives, but I am not an insider among the reindeer husbandry Sámis, due to that I grew up in a Sámi fishing–farming family.

### Insider–Outsider

In this study, the experiences, interests and knowledge of Sámis and other indigenous peoples who attend the festivals are focused on. However, as a researcher, I shifted between having an insider and having an outsider perspective. I am a coastal Sámi, educated in the Norwegian school system and have not learned to speak my parents' language/my mother tongue Northern Sámi fluently. It means that I was an outsider when I interviewed Sámi speakers and indigenous guest peoples, and the dialogue had to be carried out in Norwegian or English. At the same time, I was an insider with cultural knowledge of the region and broad competence about the coastal Sámis, as well as I have lived experienced about the diversity of Sámi people and other peoples living in the region. I grew up in the same village as the *Riddu Riđđu Festivala* is organized and know the festival and the cultural complexity (Eriksen, 2002) in the area very well. An advantage was my knowledge about the past in the region with the festivals used as tools in the revitalizing process of Sámi cultures (Hovland, 1999; Viken, 2011). This might have contributed to more intuitive understanding of the studied phenomenon, and being equipped with “insider” knowledge has given me the necessary trustworthiness among the informants and was an advantage in the access process. This also made it possible to ask the right questions and to avoid some pitfalls when I interviewed the participants.

One example where I could use my exclusive insider knowledge about the challenging past was when the old woman said to me: “I am sorry for that I am crying, but for me this is challenging to speak about. When you write about this in the “school world,” will you then speak about me and my lack of education, or will you speak on behalf of me? And, what is in it for me, will you send me the book, when it is finished?” The response from me was holding the crying old woman, and later on, I read the written text to her, and when we met for the second interview 2 years later, I gave her a book with a chapter published in Norwegian, which her grandchildren could read for her. This means that this is a bottom-up study where the researcher has to show awareness in presenting the voices of people who has been marginalized in the past (Olsen, 2016). On the other hand, the objectivity in the interpretation of the data material could be impaired. In line with Wadel and Fuglestad (2014), I did fieldwork in my own culture, which means challenges and possible pitfalls. Through “field knowledge” (Malterud, 2017), an immediate understanding of the phenomenon occurs that outsiders might not sense. Simultaneously, there is a danger of “field blindness” (Malterud, 2017) when searching for observations, which confirm one's own experiences and preconceptions. Therefore, crucial or alternative interpretations and conclusions might be overlooked, which might reduce the trustworthiness of the knowledge.

My lenses are defined by my knowledge, and as an insider, I have access to some knowledge, and when I am an outsider, I have access to other knowledge. Truth is multiple and subjective, and as a researcher, to be integrated in the research process with my family's lived experiences gave an advantage with access both to the empowered peoples and to the marginalized voices. There is no question about what is “right” or “wrong,” “true” or “not true,” but only what kind of knowledge is produced in different contexts. A systematic alternation between closeness, consideration, and critical reflection has therefore been crucial in the analytic process (Malterud, 2017; Thagaard, 2018).

### Ethical Considerations

The study proposal was guided by the Norwegian Center for Research Data (NSD), and the project followed the ethical guidelines by The Norwegian National Research Ethics Committees (2019) and NESH (2019), and an ethical approval is given from the university's ethics committee. Data analysis has been carried out in accordance with the criteria of trustworthiness and of credibility, transferability, dependability, and confirmability (Henderson, 1991, pp. 134–138). Information letter and application to carry out the study was sent to the festival organizers, and the organizers answered with a written consent. All the interviewees have read the information letter and gave their informed consent. To participate in the study was voluntary, and the participants were informed that they could withdraw from the project at any stage without consequences and get the data from their interviews deleted. For instance, both before and during the interviews, a free dialogue between the children and their parents/guardians is created to address credibility. To meet the same families several years is a strength to increase the trustworthiness, and I provided rich descriptions of the festival context to emphasize transferability. A key principle for research in national and institutional ethics in research is to keep the participants anonymous, and the participants were guaranteed confidentiality and anonymity. To ensure confidentiality, numbers and codes are used, and to protect their anonymity, the informants have been given fictive names, date and the year have been withheld and their exact roles in the festival are concealed. All potential information that may identify individuals is left out, deleted or categorized into codes and sub-codes and concealed in the text. The adult participants have read the quotations and parts of the transcribed interviews, and parents/guardians and children together have accepted the quotations used for publications. Nobody except the researcher has had the access to the interviews and list of participants, and names and numbers were kept separate and in accordance with the ethical guidelines.

### Data Analysis

The field notes and the transcribed interviews are analyzed in an interpreting and hermeneutic perspective based on the supposition that knowledge must be understood in relation to the personal and cultural contexts. Thematic analysis is used in identifying and reporting patterns in main themes and sub-themes and in analyzing the transcribed text from the interviews and observations (Braun et al., 2014). Table 1 shows examples in how the meaningful units were coded and how they were sorted in sub-themes and main themes during the analyzing process.

**Table 1:** Examples of the analysis process.

**Meaningful unit**	**Code**	**Sub-themes**	**Main themes**
“I love to hammer dry-fish to make it eatable, Heikas' grandpa taught us how to make a stallu mask.” “We speak and look differently, but we learned to make a bonfire both for winter and summer, we put up the lavvu together, and we grilled meat together at the same bonfire.” “To be outdoors every day is fun, and I like to be in the nature and learn skills and Sámi names of the places in the valley.” “We learned to build a Sámi goathi, and we always put up and furnish the traditional lavvu at Riddu.”	Type of outdoor activity offered to children and youth	Sámi outdoor life Indigenous outdoor life Learning of skills	Physical activities and outdoor life
“… when we offer activities from the guest peoples we shape and teach ‘indigenous hearts’ to children and youth.” “To bring indigenous guest peoples or ‘the Northern people of the year’ to Manndalen is crucial. They teach the children their activities, and the children increase skills and knowledge, which extend their Sámi and indigenous heart through participating in the outdoor activities at the Children's festival.” “I am proud of being a Sámi, and I love to throw lasso with the Inuits as well as to take part in the Inuit Games.” “The Native games from Russia was really tough and funny, and all the materials we used were from the nature.” “We moved, played in nature and learned to take care of new friends and the nature, and we recycled everything.”	Aims and meaning of activities offered to children and youth	Education and knowledge across generations Togetherness and friendship across cultures Environmental awareness	Indigeneity, cultural understanding and community “*The Northern people of the year*”

Through the process of the thematic analysis, four overarching themes were identified. The following headlines are presented from the findings at the festivals:

1. *symbolic capital through Sámi (doallastat, lávostallat, stallu, and goatsuballu) and indigenous outdoor activities;*
2. *Sámi and indigenous festivals—making indigeneity stronger;*
3. “*The Northern people of the year” —creating cultural awareness; and*
4. *environmental awareness through “Miljøfyrtårn” and the “Indigenous Climate Camp*.”

### Findings and Discussion

Here, I will present and discuss the findings from the study in the four parts mentioned above. The following eight Sámi festivals in Norway are studied: *Easter festival in Kautokeino* (includes the Sámi Melody Song Contest), *Easter festival in Karasjok, Riddu Riđđu festival, Márkomeannu, South Sámi Culture Festival, Sámi Week in Tromsø, Alta Sámi Festival*, and *Julesàme vahkko/Lule Sámi Week*. The *Riddu Riđđu festivala* will be presented and discussed more in detail, because at this festival, interviews, and observations were carried out with children and youth, which are the focus in this manuscript.

### Symbolic Capital Through Sámi (*doallastat, lávostallat, stallu*, and *goatsuballu*) and Indigenous Outdoor Activities

The activities offered to children and youth are numerous, and *doudji*/handcraft, sports, outdoor life, and outdoor physical activities are the main activities for children and youth at the indigenous events studied through the fieldwork. As Table 2 shows, some of the activities are similar to the work in reindeer husbandry, fishing, small scale farming, herding, harvesting, or hunting, i.e., lasso throwing, making of tools, dry-fish hammering, reindeer sledge race, or rowing the Nordland boats. Others are modern sports (i.e., football, skiing, orienteering, snow mobile races, and reindeer races), and others again are old Sámi or indigenous activities. Examples of the last types are the old Sámi ball game *goatsuballu*, making of the *stallu* (the Sámi troll) masks, preparing for the last days mask parade, several activities around the fire, *doallastat* (Sámi), or participating in reindeer sledge race. Outdoor activities are crucial in the Sámi cultures independent of if you grew up inland, in the forest or along the coast (Solbakk, 2006). In summary, outdoor activities independent of weather with close connection to nature are included in all the studied festivals. For instance, at Riddu Riđđu, children stay outdoors all day long, except from at some shows and activities carried out in the school's sports hall, in the Nis'ga Longhouse (a gift from the Nis'ga people from Canada in 2009, when they were “the Northern people of the year”), or in the different indigenous tents (*lavvu*, tipi or yurt).

**Table 2:** Activities offered to children and youth at eight Sámi festivals in Norway.

**Festival name**	**Examples of activities offered to children and youth**
Riddu Riđđu Mánáidfestivála	“*Lávostallat*” /Lavvo-ing and *doallastat*/” Bonfire-ing”, playing *goatsuballu* (old Sámi ball game) Outdoor cooking and outdoor activities for food supply Knowledge and skills in making different tents (*lavvu*, tipi and yurt) and traditional Sámi cabins (*goathi, beallegoathi*, and traditional *lavvu*) in traditional and environment-friendly ways with local wood *Duodji* (Sámi handcraft), making of tools for outdoor activities Hanging of grass, fishing, dry-fish hammering, horse-riding of local horse breed, and rowing of Nordland boats Hiking and learning Sámi names of places in the valley Making of New Year's Eve *Stallu* (Sámi trolls) masks and walking in the *stallu* mask parade on the last festival day Different outdoor activities from “the Northern people of the year” Courses in Sámi language, *yoik* (the Sámi song form) courses, animals, plants, and herbs in the North
Easter festival in Kautokeino Easter festival in Karasjok	Lasso throwing at the visor of a reindeer Reindeer race and reindeer sledge race Snow mobile races Skiing—cross-country skiing *Doudji*, making tools for outdoor life *≪Gávvilis rieban≫* (The smart fox) Different outdoor physical activities Course in yoiking (the Sámi song form)
Márkomeannu South Sámi Culture Festival Sámi Week in Tromsø Alta Sámi Festival	Lasso throwing at the visor of a reindeer Course in Sámi language and culture Course in yoiking and harvesting from nature Sámi authors, seminars, and exhibitions Different forms of physical outdoor activities like ≪Vætna≫ *≪Lávvotek≫* = discotheque in a *lavvu Doudji* and Sámi handcraft traditions Rievvarnieida Reindeer race and reindeer sledge race Sámi mode photo course
Julesàme vahkko/Lule Sámi Week	≪Nature as playground≫ along the coast in ebb-tide Orienteering in nature *Doudji*, making tools for outdoor life Football *Lávvo*-evening

Together with the volunteers and staff members, great-grandparents, grandparents, parents, museum employees, local sports clubs or handcraft/*doudji* institutions (husflidslag), who have the competence and still know the skills, teach the children and youth. In similar ways, indigenous peoples from other countries contribute *via* “*the Northern people of the year*” projects, when they present their play and games and involve the children at Riddu Riđđu. Each year, a new group of indigenous peoples are guest peoples at Riddu Riđđu as “*the Northern people of the year*,” which is elaborated further later on.

A recent study about youth activities in Northern Norway shows that Sámi-speaking youth are more active outdoors than their Norwegian-speaking counterparts, and they keep alive the harvest and gathering traditions, and especially hunting and fishing are popular outdoor activities among the Sámis (Rafoss and Hines, 2016). However, the festival leaders state that the grandparent generation's role is of great importance in transferring Sámi outdoor skills and knowledge or cultural capital (Bourdieu, 1996) to the children and youth. Nowadays, most Sámis work in ordinary jobs, and a lot of skills and knowledge outdoors and in nature are lost, i.e., the thousands of Sámi words for snow, reindeer husbandry skills, reading of and preparing for different kinds of weather, different kinds of birch-wood for use in building of *lavvu, beallegoathi*, and *goahti*, and fishing and hunting skills. Therefore, the organizers said that “elderly people act as ‘pathfinders’ or teachers” at all the studied festivals. Through the teaching of outdoor life, the cultural knowledge is transferred from elders to newer generations, which might enrich children and youth in form of embodied knowledge and develop their cultural habitus.

The children at the festivals have variable knowledge about and skills in the festival activities, dependent on being indigenous or not, if they are from inland, coast, forest or urban areas, and dependent on family upbringing. A diversity of activities are taught, i.e., drying of meat or fish as is common food preservation in all Sámi cultures; lasso throwing at the visor of a reindeer, which is used in reindeer husbandry; coastal Sámi traditions like hammering of dry fish to make the dried fish eatable (*tørrfiskbanking*); *stallu* mask making for a parade, which still is going on in Olmáivággi/Manndalen on New Year's Eve; and *goatsuballu*, a traditional ball game with sticks and ball. Children and youth enjoy both traditional and newer activities and comment especially about the enjoyment of the traditional Sámi activities:

I love to hammer dry-fish to make it eatable, Heikas' grandpa taught us how to make a stallu mask. (“Jotta,” 8)

I am afraid of stallu, but I like to hammer dry fish and eat it afterwards. (“Janna,” 5)

To be outdoors is fun and I like to be in the nature and learn skills. We learned to build a Sámi goathi, and we always put up and furnish the traditional lavvu. (“Laura,” 10)

In addition to dry-fish hammering, *stallu* mask parade and learning to make Sámi *goathi* or traditional *lavvu*, some children enjoy the rowing of Nordland boats, and for other children, the most fun activity is “to ride the Lyngen horse along in the forest and along the river” (“Milda,” 5). As mentioned, the children stay outdoors during three full days at *Mánáidfestivála*, because “Sámi children shall learn how to stay outdoors independent of weather” (Mother), in line with the traditions in the North. The children also reflect upon being outside the whole day, and “doing exciting things in nature,” and most children enjoy moving around outdoors instead of “play computer games at home” (“Jotta,” 8).

As mentioned in the *Introduction*, all Sámi languages use active words about doing things in nature. Two examples of such language knowledge or cultural capital (Bourdieu, 1996) are “*lavostallat*” about activities connected to the Sámi tent, including furnishing the *lavvu* and doing activities inside and outside the *lavvu*. Another example is the use of the fireplace, which is of great importance for Sámis and other indigenous peoples. To make a bonfire in Northern Sámi is “*doallastat*” (“bonfire-ing”), which includes to make a bonfire as well as working or making other activities around the fire, such as cooking or preserving food, making coffee, making “*duodji*” (Sámi handcraft), keeping predators or mosquitos away, lightening up the area in the dark time, or just relaxing and talking. When the children were taught “*dollastat*,” “*lavvostallat*,” and “*doudji*” to make tools, i.e., wooden sticks, some children use a big knife for the first time. Several of the skills in nature previously were common in all households, but today, the festivals seem to be crucial tradition bearers. This is embodied knowledge for the Sámi elders and new cultural capital for children and youth at the festivals. The old Sámi view on nature is that nature is “not human-made,” and the culture is “human-made,” and several Sámi gods and goddesses in the old Sámi religion were crucial in the meeting between nature and culture and still are for many Sámis. For instance, at the festivals, stories were told to the children about the Sámi goddess *Sáráhkká*, who had her abode at the field and was sitting at the end of the fireplace, and she always got a part of the food.

To respect nature is crucial in Sámi/indigenous culture, and a part of it is to save nature for future generations. The Sámi elders involved with teaching of the children at *Mánáidfestivála* underline the importance of transferring cultural capital as outdoor life and the harvesting culture to the children. The combination of care for nature and use of nature for harvesting and food-supply is elaborated by the elders. “Petra” stated that “Sámi do not go for ‘hiking only’ in the nature, due to that the activities outdoors in nature must be useful for the household.” Examples that came up in the interviews are that walking in nature is a combination of, i.e., searching for cloudberries; herding of reindeers; collecting sheep; bringing of food, water, and salt stones to the animals; hunting; and fishing, which also have similarities with activities of other people in Norway living in the rural areas.

Many places in the village Olmáivággi/Manndalen do only have names in Sámi. During the hiking in the valley, the children were taught the Northern Sámi language and shown several holy places in nature from the old Sámi religion, for instance, a mountain (i.e., Manndalshodet, Storhaugen, and Kjerringdalsfjellet) or a stone (i.e., Storsteinen and Offersteinen), and one of the Sámi elder involved with the children's activities said:

The children shall be taught traditional knowledge and experiences and learn skills as our forefathers and foremothers used in everyday life, like doallastat, lavostallat, making of goathi and beallegoathi, learn to use the material from the local forest and be environment-friendly and learn to show respect for the nature. They have to learn it here at the festival, because the Sámi knowledge is not taught in schools and in modern families.

Children and youth are introduced to such skills outdoors and in nature at the Sámi festivals, including knowledge of the fauna and flora in the North, the Sámi words of nature and places, the knowledge about the traditional *lavvu* and how to survive outside and in nature the Sámi way. The Sámi tent, *lavvu*, is similar to the tipi, and the *lavvu* is crucial in Sámi outdoor life. Today, the use of light-weighted *lavvu*s are common in outdoor life all over Scandinavia. Other kinds of Sámi cabins are the *goathi* and the *beallegoathi*, which only might be taught by those with knowledge about the handcraft, including which type of strong birch-wood to use and which tools are useful for making these cabins. “You cannot learn about this in books, you must get out in the nature and learn it by making it,” as “Biret” said. Here, the governing bodies of the festivals focused at the aims to “give unique Riddu Riđđu-experiences with doudji and teaching of Sámi handcraft which is useful both indoors and outdoors” (interview, festival leader). The local museum Nord-Troms Museum and the handcraft organization “Husflidslaget” in Manndalen were involved in the teaching. Festival staff underline the importance of teaching Sámi and indigenous skills and ways of living to the children, and especially the lost coastal Sámi knowledge and skills are elaborated on:

We have to teach the youth Sámi and indigenous skills and ways of living, because most knowledge is taught by word-of-mouth from generation to generation, and else the knowledge is lost forever. Coastal Sámi knowledge and skills are especial essential here in this coastal area, where the assimilation has been strong. (Interview, festival staff)

The festival activities offered to children and youth might be defined as crucial symbolic capital, and cultural capital in all three forms (Bourdieu, 1996): firstly, they are shared through the embodied knowledge of Sáminess taught by the elderly Sámis. Secondly, they are objective compositions as knowledge of type of birch-wood and type of tools to make *goahti*s, *doudji* and Sámi/indigenous art and skills used in nature. Thirdly, they are more institutionalized compositions as Sámi work titles and different Sámi languages as well as the Sámi song form *yoik*. In addition, they are field-specific capital with a here and now festival experience together with other indigenous and non-indigenous peoples.

### Sámi and Indigenous Festivals—Making Indigeneity Stronger

The findings show that all eight festivals studied have for a long time exhibited and worked to strengthen pride in Sámi culture. Musical, cultural, and outdoor activities and performances are held at all festivals. In 2021, *Riddu Riđđu festivala* (Riddu Riđđu, 2021) has 133 volunteers, 120 voluntary staff members, four full-time employees, and one working on a project to develop the festival. As an example, Riddu Riđđu organize a 5-day programme with 80–90 arrangements during the festival week every year. This festival has a year-round programme, and in addition to the programme during the festival week in July, 20 single arrangements (concerts, performances, courses, seminars, paper presentations, and lectures) are organized during the year locally, regionally, nationally, and internationally. For adults, the offers of activities are many with concerts by a numerous Sámi and indigenous artists, films, several workshops, Sámi language courses, yoiking courses, exhibitions, and seminars with presentation of recent research in collaboration with universities, presenting theater and dance performances, art, “*doudji*” /handicrafts, and physical and outdoor activities.

All the eight events are collaborating with other national and local institutions in programming and implementing of the festival. Examples are local authorities in the region and the governing bodies in the respective municipality, schools, universities, i.e., UIT-The Norway Arctic University and Nord University, museums; music, art, and handcraft organizations; Sámi language centers; and sports clubs. There is also collaboration with other events like Buktafestivalen (Tromsø), the Sámi Melody Song Contest (Kautokeino), the Midnight Sun Marathon (Tromsø), Naturvernforbundet at Riddu Riđđu (Manndalen), and the Sámi Fashion Week (Alta). Viken (2013) stated that the development of Riddu Riđđu has made this festival one of the most significant international indigenous festivals in Europe. My findings are also in line with those of Jæger (2019), who underlined the importance of the festivals as meeting places, and the benefit for local communities in Northern Norway. The growth and clear focus at revitalization of Sámi culture and identity have led to visibility and media interest across borders. As examples, international media from Russia, the USA, Canada, Australia, New Zealand/Aotearoa, and Taiwan have broadcasted in TV, social media, and written press from the festivals.

Riddu Riđđu organizes *Nourat* for young people below 18 years old, where Sámis and other indigenous and non-indigenous peoples meet and develop a performance together, which is shown at the main stage during the festival week (Riddu Riđđu, 2019a). Some statements from the participants at Nourat might show the path in the answers from the youth:

I came out as Sámi thanks to this festival, before I took part in the Nourat with Sámis and indigenous youth from many parts of the world, I never dared to show my Sáminess in public. (Male, 17)

I was ashamed of being a Sámi before, when I was younger, and our identity was a kind of family secret, which we did not talk about neither at home nor outside home. Today, after being involved in Riddu Riđđu, I always wear any kind of Sámi symbol, for example the scarf, jewelry with Sámi symbols, the luhkka (a Sámi poncho) or doudji (Sámi handcraft) products. Take a look at my ear rings with the “*riebangardi*” (Riddu Riđđu symbol), she said when smiling proudly. (Female, 15)

It was at Riddu Riđđu I saw other youngsters from Manndalen was wearing the Sámi dress with pride. This helped me to get self-esteem enough to ask my family to buy one for my confirmation celebration, and now I wear my beautiful “Lyngenkofta” (coastal Sámi dress) at any celebrating occasions. (Female, 16)

A 3-day-long festival for children between 3 and 13 years old called *Mánáidfestivála* is organized yearly at Riddu Riđđu since 2001. The programme takes place outdoors and includes a maximum of 120 children each year. Children of below school age are required to be accompanied by an adult to assist them in the activities. Skogvang and Trollvik (2018, p. 46) emphasized the importance of the festivals for indigenous peoples globally, as the festival might result in that “… prouder youth with their heads held high can proclaim that they have a Sámi background.” Skogvang (2020, p. 10) showed that Riddu Riđđu in Olmáivággi has become “… an annual meeting place for relatives who live in different parts of the country and in other countries.” The children underline the importance of *Mánáidfestivála* at Riddu Riđđu for them like this:

I am proud of being a Sámi and I love to throw lasso with the Inuits as well as to take part in the Inuit Games. (Jussi, 8)

Mother helped me with the knife when I was making the wooden stick for grilling of meat and sausages, and we learned Sámi language and yoiking inside the lavvu both this year and last year. (Nelle, 4)

When you take part in *Mánáidfestivála* you don't have to be a Sámi, but you learn a lot about Sámis and other peoples. This year I learned circle-dancing from Suming and his gang from Taiwan. (Jenny, 7)

At home I am often sitting and gaming on the computer, but here we are doing a lot of funny things like ATV driving 3 years ago, horse-riding at the friendly Lyngen horse last year, make bonfire every year, dry-fish-hammering, and the Native Games with people from Russia was really tough and funny, and all the materials we used were from the nature. (Isak, 11)

As the data show, the children and youth are equipped with knowledge and skills of their own culture, which strengthen their indigeneity and equip them with symbolic capital (Bourdieu, 1996). Examples of how cultural capital strengthen their “Sáminess” or cultural habitus are the teaching of knowledge about and skills in the Sámi languages and Sámi yoiking, and the participants feel safer in expressing their identity through wearing the Sámi dress and other Sámi symbols.

The findings are in line with earlier research about festivals in general, which shows that festivals are important identity markers (Quinn, 2005; Jæger, 2019), and they also support earlier research about the region where the young Sámi habituses are marked by the marginalized past (Hovland, 1999), as well as the festival's importance for the revitalization of the Sámi cultures and importance for development of places (Hansen, 2007; Viken, 2011; Viken and Jaeger, 2012). In an international context, the opposition to mainstream society and engagement in nation building are shown by several researchers (Bale and Cronin, 2003; Mangan and Ritchie, 2004; Hallinan and Judd, 2013), and this study shows similarities to their findings. In the same ways as international events as *North American Indigenous Games* and the *World Indigenous Nation games* (Paraschak, 1997; Forsyth and Wamsley, 2006; King, 2006; Hallinan and Judd, 2013) are used in revitalization processes, this study show how the meetings at the studied Sámi/indigenous festivals are used in the participants' Sámi revitalization processes with creating stronger cultural habituses through experiences of empowerment instead of focusing on the marginalized past.

### “The Northern People of the Year” —Creating Cultural Awareness

Since 2000, Riddu Riđđu has brought the international meetings among indigenous peoples further, calling attention to various indigenous peoples at the festival as “*the Northern people of the year* (Riddu Riđđu, 2011).” Each year, a new group of indigenous peoples are guest peoples at the festival, and they present their culture through activities, performances, concerts, art, handcraft, food-tasting, etc. They come from different continents and perform at the adult festival as well as have workshops for and together with the children and youth at Riddu Riđđu. Examples are the *Veps*, a Finnish-Ugrian people living in Karelen in Russia (2010); *Ami, Atayal*, and *Paiwan* from Taiwan (2016); and Eastern Sámis living in Russia (2018). The festival directors describe the aim of the visiting peoples like this:

An important aim with the “Northern People of the Year” program is to let our audience learn from the delegation. Through their workshops for children and youth, they give our youngest participants an understanding and appreciation of indigenous peoples' knowledge and cultural practices. (Festival director, female)

Our guest peoples brings the international world to this little village Manndalen, and through the meetings between Sámi children and youth and indigenous peoples from for instance, Canada, USA, or Russia they get knowledge of other indigenous peoples with similar background as ourselves. (Festival director, male)

My wish is that the children will extend their Sámi and “indigenous heart” and knowledge and skills from different peoples through taking part in *Mánáidfestivála*. We want them to be tolerant and inclusive. (Festival director, female)

Different forms of outdoor activities and sports have increased during the sharing from other indigenous guest peoples, and in Bourdieu's (1996) terms, these exchanges with visiting peoples create both social and cultural capital. The visiting delegations are financed from Riddu Riđđu and governing bodies in Norway, coordinated from both Norway and the visiting countries, and the indigenous artists themselves curate the programmes and workshops. Some examples of activities from “Northern People of the Year” are the “Hoop dance” workshop with James Jones in 2014, dances with Six Nations from Canada in 2015, “Alaska Native Games” in 2017, “East Sámi Games” in 2018 or “Inuit Games” from Nunavut in 2019. Another example is from 2016 when Riddu Riđđu invited a delegation of indigenous artists, cultural bearers and youth from Taiwan to participate and perform at the festival as the “Northern people of the year.”

The festival organizers chose to focus on the three largest groups of indigenous peoples from Taiwan: the *Atayal, Paiwan*, and *Amis*. *Sakinu Ahronglong* is Paiwan and leads the Youth groups and the organization called *Hunter School*. The Hunter School was established in 2004 and is centered in the village referred to as “*Lalauran*” in Paiwan language, in the South–East County of Taitung. The Hunter School was established as an alternative for girls who were interested in socializing and gaining traditional knowledge in a similar manner as how the Paiwan Youth groups for boys function. The basic explanation given is that it functions as a gathering point for young people who seek knowledge on how to interact with and in nature, guided through the Paiwan ethos. Sakinu especially was invited to Riddu Riđđu to talk about how he educates practical knowledge of hunting, aiming to empower young people on their abilities in nature and in that way strengthen their self-worth. Sakinu also brought a group of Hunter School members, who volunteered at the festival. The participating children and youth enjoyed circle-dancing with Suming, and the young boy “Jotta” said that “The Hunter-school activities last year was one of my favorites.”

In Bourdieu's terms, symbolic capital (1996) from other indigenous peoples might strengthen Sámis' habitus (Bourdieu and Wacquant, 1992); due to that, participation at Riddu Riđđu is experienced as empowering. Both Sámis and other indigenous peoples experience empowerment when they are performing separately or together at the safe environments at the festival stage in Northern Norway. Their knowledge and skills and their ethnicities are identified as valuable cultural capital. At these events, social capital is identified *via* establishing networks between indigenous peoples, as well as the shared cultural capital is strengthening the peoples' self-esteem and their indigeneity. A quote from an artist from Greenland shows how the sharing of activities during the festival allows them to develop cultural capital and empower their habitus:

This place and this festival create pride, love and understanding among people from the whole world. From what happens during an indigenous week in this valley, we go home to our countries and our hearts and souls are stronger, and we have much more knowledge about other people. (…) Why? Because we get the power from different indigenous cultures, which we can use positively in many settings. We understand diversity and can handle this better in a changing society. (Woman, 2014)

Riddu Riđđu is said to give power to peoples from different indigenous cultures, and a man and father from Northern America underlined how one get advantages of taking part in Sámi festivals:

The festival activities in the nature make our own “indigeneity” stronger when we go back home to our country of living, and at the same time we are Americans. We are both in many ways. (Man, 2014)

As shown, the indigenous youth at Riddu Riđđu tell that “the festival makes our own indigeneity stronger when we go back to our country of living.” This is comparable with the experiences from indigenous peoples from Taiwan, who visited Riddu Riđđu in 2016 as the “Northern people of the year:”

After a week at Riddu Riđđu, I feel empowered when I come home to my island, because the common sad history, the connection to the nature, outdoor life and the performances from our people at this festival make us visible in our home country. (Woman, 2016)

The visiting peoples from Taiwan who traveled far North to Olmáivággi/Manndalen in 2016 got extraordinary attention, because the media coverage was huge. They were followed closely from media through the Riddu Riđđu festival week and broadcasted live daily back to Taiwan on the main TV channel, as described by one man:

For us it was like freedom to be who we are, and a principal TV channel followed us during Riddu Riđđu, broadcasted the activities to a broader audience in Taiwan (…) and made me proud of my ethnicity.

Ethnic identity was presented as positive symbolic capital (Bourdieu, 1996) for many of indigenous peoples from Taiwan both at the festival in Norway and in TV on Taiwan. Because their indigenous identities were highlighted and celebrated at this festival, their diversity in indigenous identities was identified, appreciated as valuable and creditable or worthy, or defined as positive symbolic capital (Bourdieu, 1996, p. 90). Tan's study (2012) shows that indigenous festivals on Taiwan are crucial sources of local pride and great celebration of the particular indigenous traditions, which have similarities to the Sámi festivals in strengthening the communal relationships and reconnection to the indigenous identity. In line with other researchers (Quinn, 2005; Jæger, 2019), old identities are preserved and strengthened and new identities are constructed during meetings between the participants and the visiting peoples, and several forms of games might provide Sámis, indigenous, and non-indigenous participants with knowledge and skills which that similar to the Dene peoples' experiences (Heine and Scott, 1994). To put forward old traditions in new contexts as the presented festivals gives cultural importance of their old games within the context of contemporary Norwegian, Western, or European cultures.

Both staff and festival governing bodies underline the importance of the indigenous guest peoples, for instance, one festival staff said: “…, the guest people is crucial for us to create the future society.” The leaders refer to the wish to create “*indigenous hearts*” or cultural capital and that they want to create acceptance and diversity and “teach people to be proud of being indigenous” (“Janna”) for changing their habitus (Bourdieu and Wacquant, 1992) from being marginalized to experiencing empowerment. The governing bodies furthermore want to shape experiences of community and give a knowledge and understanding of different cultures and, as “Ravra-Hans” said, “… create the society of the future.”

As a participant at Riddu Riđđu, you meet different indigenous peoples, Norwegians and other non-indigenous peoples from the mainstream culture. “*The Northern people of the year*” brings both adults, youth and children to the festival; and the artists perform for and together with children at the *Mánáidfestivála* and youth at *Nourat*. The informants underline that to take part in indigenous festivals in Norway together with other people with similar backgrounds is empowering and imparts them with knowledge and skills of both minorities' and major populations' handcraft, physical and outdoor activities. The activities represent different indigenous sports and outdoor activities and seem to create sustainable ties between persons, networks, and organizations and to build identities and bridges between participants (social capital).

We speak and look differently, but we learned to make a bonfire both for winter and summer, we put up the lavvu together, and we grilled meat together at the same bonfire. (“Isak,” 9 years old)

We enjoyed the Inuit Games, and they are proud peoples in the same way as I am proud of being a Sámi. It was super-funny to teach our traditions with eating dry fish and teach them how to make dry-fish eatable with a hammer. (“Peder,” 12 years old)

Through offers of activities and workshops for youth at *Nourat* and children at *Mánáidfestivála*, Sámis, non-indigenous peoples and the visiting indigenous peoples give the youngest participants an understanding and appreciation of indigenous peoples' knowledge and cultural practice (cultural capital), which might change the embodied knowledge (Bourdieu, 1995) that resulted from the “Norwegianization process.” To express Sáminess and other indigenous identities is pinpointed as being vital by children, youth, and also their parents/guardians who take part in the festivals. Several indigenous games and plays are offered to the participants at the Sámi festivals, i.e., lasso throwing competitions, snow mobile competitions, reindeer races (Sámi Games) or games from other indigenous peoples at the Sámi festivals: the Inuit Games, Native Games from Russia or the Kildin Sámi or Eastern Sámi Games. In line with Heine and Scott (1994), these games at the Sámi festivals are shown the cultural importance similar to the Denes' old games within the context of contemporary Western culture.

### Environmental Awareness Through “Miljøfyrtårn” and the “Indigenous Climate Camp”

During the last 15 years, all festivals have put focus on increased environmental awareness, and this is especially noticeable at the Riddu Riđđu festival. Riddu Riđđu has for a long time put focus at developing an environment-friendly festival and was one of the first festivals in Norway that in 2004 were certified as “*Miljøfyrtårn*” or “green/environment-friendly” event (Riddu Riđđu, 2011). The festival was re-certified in 2019, and each year, the festival “wants to take steps to reduce environmental foot prints” (festival leader). The governing bodies express a clear policy in promoting environmental sustainability for the festival:

Riddu Riđđu Festivala AS has an objective to become a sustainable festival organization with good resource utilization and a minimum of negative influence on human beings, nature and milieu. (Riddu Riđđu, 2011, 2019b)

The oldest environmental and nature protection organization in Norway is Naturvernforbundet/Friends of the Earth Norway (2021). This organization's aims are

… to strive to protect nature and the environment in such a way that human activity does not exceed the carrying capacity of nature, and work for a society where people live in harmony with nature. (…) This is a society where the basis and diversity of life is secured for future generations, and where the nature's own values are the foundation of the work to increase man's respect for, and love of, life and landscape.

In 2019, same year as Riddu Riđđu was re-certified as a green festival or “Miljøfyrtårn,” the festival asked Naturvernforbundet to participate at the festival and to control how environmental-friendly Riddu Riđđu had become. At the same time, Naturvernforbundet was asked to give advice on how the festival could improve even more. The conclusion was that:

The festival has done a great effort to increase the environmental-friendliness and to inspire people to live in harmony with nature, following natures' values and increase the human beings respect for the nature. Examples especially mentioned were public transport with buses to/from the festival area and environment-parking fee for cars and vans, increased recycling and increased use of environment-friendly equipment, use of locally produced food and products, re-useable milieu-friendly brand products like t-shirts, hoodies, cups etc., increased recycling of plastic, paper, cans, glass and other materials.

Despite all the positive factors and the re-certification, the study by Naturvernforbundet recommended the festival to improve the following factors (Riddu Riđđu, 2019b):

Even better recycling of plastic, more coordinated transport of artists to/from the festival, milieu/climate-hosts and climate information-lavvu are recommended at the festival area.

In observation of the activities at *Mánáidfestivála* and *Nourat*, the effort for environment-friendly festival is visible in several ways. Children and youth are served food and drinks in cups and plates that are recyclable or recycled, and they are encouraged themselves to practice recycling. There are several dust bins available that are clearly marked for plastic, paper, food waste and general waste. The volunteers and staff teach the children recycling at the same time as they explain what to do and why they have to do it. Children and youth explain their experiences about skills in recycling and climate-friendly activities, as follows:

I and my friends have learned to survive outdoors in traditional climate-friendly ways.

My sister and I have told mam and dad to recycle more and to drive less car. Here we walk to and from the festival area from the family camping organized by the festival.

Other citations focus at the closeness to nature in both Sámi and other indigenous cultures and that they have been taught skills in how to survive outdoors in traditional ways and how to build old Sámi homes and houses, i.e., the traditional *lavvu, beallegoathi*, and *goathi*:

We cook, make food, eat dry-fish and do not need all the waste that we do in everyday life. Last year we learned how to build a beallegoahti and yearly we have put up and furnished one traditional lavvu. In addition, we have been walking in the valley learning Sámi names of the places, and how grandma lived. To stay close to nature gives good feelings, so I will try to continue with this, and we will come back to the festival.

We should have lived more as the old Sámis did or as the San people from Africa do, who lives closer to the nature.We have learned to survive outdoors during all 3 days.

As the citations show, the children appreciate staying outdoors, be in nature and learn skills in nature, including climate-friendly activities.

The Sámi youth highlight that a global network with other indigenous peoples is established after meeting each other at the Riddu Riđđu festival. The social gatherings have resulted in addressing climate challenges at the festival seminars as well as outside the festival. Creation of sustainable societies is of great importance for indigenous peoples across the globe, and one example that got large media attention is the fight and the protests against the Dakota Access pipeline in Standing Rock in the USA. The collaboration between the Riddu Riđđu employees, *Nourat* and other Sámi youth organizations has resulted in planning of a global *Indigenous Climate Camp* (Riddu Riđđu, 2020). The objectives for the event are to put environmental issues on the agenda from Sámi youth perspectives and gather Sámi and indigenous youth at the festival in a climate workshop. At the workshop, the plan is to create a climate manifest and communicate this *via* social media, video, and films and collaborate with indigenous peoples from other continents, i.e., the USA and Canada. The programme is financed by the Riddu Riđđu festival and should have been held in 2020 at the festival area, but it was postponed to 2021 due to the COVID-19 pandemic. The festival documents tells that when the society opens up again “indigenous youth from all over the world will meet at the festival in Olmáivággi to share their experiences and increase their environmental awareness and gain knowledge and skills to both care for human-beings and nature in the future.” In the planned programme, it is stated to be locally planned at the same time as it brings the Sámi youth perspective on climate to the global stand.

As mentioned, symbolic capital (Bourdieu, 1996) is what social groups recognized as valuable. In relation to the studied festivals, the established networks between indigenous peoples in Norway and worldwide in collaboration on environmental issues might be defined as social capital. To learn skills and gain knowledge about Sámi outdoor activities, which also are closely connected to nature and outdoor life and learn about Sámi/indigenous cultures at the festival, equip the youth with cultural knowledge and skills or cultural capital, including how to protect nature for future generations. That people take part in joint activities, for instance, outdoor activities in nature, at the same time as they are extra receptive of different kinds of perception, might make the elements in the activities more embodied. Through presence in time and place with strong thematic focus, as at the Riddu Riđđu festival and the other Sámi festivals are, opens for a strong feeling of community among the participants through tasks and experiences (social and cultural capital). That the participants steadily have to conduct themselves to new contexts might lead to changes in their habitus (Bourdieu, 1995; Bourdieu and Wacquant, 1995), which gives space for new thoughts, creativity, and multiple identities. The new thoughts might be exemplified with the planned *Indigenous Climate Camp*, where the young indigenous peoples who met at the Riddu Riđđu festival have invited indigenous youth from several parts of the world to gather and develop a climate manifest.

In summary, these findings show that to a certain extent the festival participants, performers, and organizers have developed their environmental awareness through being involved in the festival. Cultural awareness has been in focus through all festivals, but the last 15 years more focus is put on creating a sustainable society and climate for future generations. The governing bodies are not satisfied with being a “Miljøfyrtårn” only, which means that they are doing well in increasing environmental awareness. They want to increase the effort, and the future will show how the networks between indigenous youth around the globe might succeed with the objectives that Riddu Riđđu has outlined for the Indigenous Climate Camp.

## Conclusion

The indigenous festivals have a crucial role in filling the gap in knowledge about Sámis and other indigenous peoples. The experiences of the discrimination through the “Norwegianization process” (Skogvang, 2019) are an “embodied knowledge” (Bourdieu, 1995) in Sámis of today in line with Hovland (1999), who stated that young peoples' cultural habituses are characterized by the discriminating history from the mainstream society. Experiences of discrimination and experiences of painful assimilation are deeply rooted in and fundamental to the habitus (Bourdieu and Wacquant, 1995) of the young Sámis in the region (Hovland, 1999) as it is for indigenous peoples at other continents (Paraschak, 1997; Forsyth and Wamsley, 2006; King, 2006; Hallinan and Judd, 2013). Nevertheless, in line with international research (Bale and Cronin, 2003; Mangan and Ritchie, 2004; Quinn, 2005; King, 2006; Hallinan and Judd, 2013; Jæger, 2019), the study shows that the festivals studied have qualities, which might contribute in shaping Sámi and indigenous identities and cultural lifts in the local communities (Phipps and Slater, 2010; Tan, 2012). This study supports earlier research about Sámi festivals in Norway where the festivals are leading spaces of innovation in creating a sustainable, secure, and mature national culture (Jæger, 2019), vital for local peoples' revitalization process (Pedersen and Viken, 2009) and gives cross-cultural recognition, respect, exchange, and creativity (Viken and Jaeger, 2012; Viken, 2013).

The informants highlight that to take part in indigenous festivals in Norway together with other people with similar backgrounds is empowering and imparts them with knowledge and skills of both minorities and the major populations' cultural and outdoor activities. Participating at the festivals equips youth, children, and their parents/guardians with several forms of symbolic capital (Bourdieu, 1996). Through the different indigenous activities, ties between indigenous peoples worldwide established provide participants with social capital, and they give indigenous artists worldwide a safe arena to perform at and gain economic capital when performing at the festival stand. In addition to the international networks (social capital) and visibility, the festivals influence the economic development in the region and municipalities they are situated in Viken and Jaeger (2012), Skogvang (2013, 2016), Viken (2013), and Jæger (2019). The involvement might create what some informants describe as “*indigenous hearts*.” This is an aspect that can create social change and an alternative future for indigenous peoples and increased understanding from non-indigenous peoples taking part in the festivals. To meet different indigenous peoples, outdoor activities in nature together in new and safe contexts might lead to habitus transformation (Bourdieu and Wacquant, 1995), from painful history to increased self-esteem and empowerment among indigenous peoples.

In addition, the activities represent different indigenous physical, cultural, and outdoor activities (cultural capital) and seem to create sustainable ties between persons and between institutions, which is a valuable social capital given the sustainability for the future. One example of extended social capital is the networks established between organizations, artists and governing bodies, which have formed the “Indigenous Climate Camp.” Due to the experiences of the participants in this study, I conclude that to participate at Sámi/indigenous festivals is empowering and adds knowledge and skills about Sámi and indigenous culture and societal life, which might create environment-friendly and sustainable societies and cultures. The festivals add cultural knowledge and skills lacking in other institutions, and the environment-friendly outdoor activities might be continued through such knowledge and skills. This is not a field-specific capital and a happening here and now only, because of the huge collaboration across institutions and because the knowledge and skills learned at the Sámi festivals are transferred to other institutions at language centers, schools, and international youth organizations, and the information is spread internationally *via* TV, radio, social, and other media.

The participants are equipped with cultural awareness through the festivals as a space for meetings between different indigenous and non-indigenous peoples, and the children and youth are inspired to enjoy the life in nature, caring about the environments and respecting nature. The planned Indigenous Climate Camp with focus on climate from a Sámi youth perspective is locally situated at the same time as it is programmed to move to a global stand with creating a climate manifest inspired by knowledge from indigenous peoples from other parts of the world. Anyway, more research is needed to study how the children and youth have increased their cultural and environmental awareness during taking part in the Sámi festivals, which also is outlined as crucial capital for the future in United Nations' sustainable goals.

## Data Availability Statement

The data analyzed in this study is subject to the following licenses/restrictions: The Norwegian Center for Research Data (NSD) evaluates research institutions to ensure ethical consent in research. The study proposal guided by the NSD, where the informants were guaranteed confidentiality and anonymity when they gave their informed consent. Because of ethical matters and due to privacy restrictions the data is not possible to share. This is in accordance with NSD acceptance of the project. The informants are guaranteed confidentiality and anonymity. Children below school age are interviewed together with their parents who gave informed consent. The informants given fictive names, the date and year of the interviews been withheld, and so has their exact roles at the festival, to protect anonymity. Requests to access these datasets should be directed to bente.skogvang@inn.no.

## Ethics Statement

The study proposal was guided by the Norwegian Centre for Research Data (NSD) and the project followed the ethical guidelines by The Norwegian National Research Ethics Committees (2019) and NESH (2019), which is in line with UK Research Integrity Office – Association of Research Managers and Administrators, and an ethical approval is given from the university's Ethics Committee. Data analysis has been carried out in accordance with the criteria of trustworthiness and of credibility, transferability, dependability, and confirmability (Henderson, 1991, pp. 134–138). Information letter and application to carry out the study sent to the festival organizers, and the organizers answered with a written consent. All the interviewees has read the information letter and gave their informed consent. To participate in the study was voluntary, and the participants were informed that they could withdraw from the project at any stage without consequences and get the data deleted.

## Author Contributions

BS was the chair of the project Sami festivals and has carried out the research from start until the published material.

## Conflict of Interest

The author declares that the research was conducted in the absence of any commercial or financial relationships that could be construed as a potential conflict of interest.
